# 

**Published:** 1856-08

**Authors:** 


### CONTENTS.

### *ORIGINAL COMMUNICATIONS.*

PAGE

ARTICLE I.—An Address, Introductory to the Second Course of Lectures in the Atlanta Medical College. By ALEXANDER MEANS, M. D., Professor of Chemistry and Pharmacy. Published by the Class, May 1st, 1856.......... 706

ARTICLE II.—A Thesis on Typhoid Fever, presented to the Faculty of the Oglethorpe Medical College, at Savannah, Ga., Session 1855 and 1856. By JONATHAN J. JONES, of Knoxville, Ga. Published by the Faculty............ 723

ARTICLE III.—Report of a Case of Shoulder Presentation. By R. T. FOOTE, M. D., of Society Hill, Ala........................................ 744

ARTICLE IV.—Vitis Vinifera Radix, as a Diuretic.............................. 746

### *SELECTIONS.*

The Musk-Deer.............................................................. 748

Observations on the Cause of the Disease known as the Sun-Stroke. By SANFORD B. HUNT, M. D.............................................................. 752

### *EDITORIAL AND MISCELLANEOUS.*

Close of the Volume.......................................................... 657

To Subscribers................................................................ 759

Medical Etiquette and Professional Rights.................................. 759

Publications Received........................................................ 762

Dry Gangrene.................................................................. 664

To those whom it may concern............................................... 764

Errata......................................................................... 764

